# Reliability and reproducibility of cardiac MRI quantification of peak exercise function with long-axis views

**DOI:** 10.1371/journal.pone.0245912

**Published:** 2021-02-04

**Authors:** Amy A. Kirkham, Michelle V. Goonasekera, Brenna C. Mattiello, Justin G. Grenier, Mark J. Haykowsky, Richard B. Thompson

**Affiliations:** 1 Department of Biomedical Engineering, University of Alberta, Edmonton, Alberta, Canada; 2 Faculty of Kinesiology, Sport, and Recreation, University of Alberta, Edmonton, Alberta, Canada; 3 Faculty of Nursing, University of Alberta, Edmonton, Alberta, Canada; Scuola Superiore Sant’Anna, ITALY

## Abstract

The conventional approach to cardiac magnetic resonance (CMR) involving breath holds, electrocardiography-gating, and acquisition of a short-axis (SAX) image stack, introduces technical and logistical challenges for assessing exercise left ventricular (LV) function. Real-time, free-breathing CMR acquisition of long-axis (LAX) images overcomes these issues and also enables assessment of global longitudinal strain (GLS). We evaluated the reliability of a free-breathing LAX approach compared to the standard SAX approach and the reproducibility of free-breathing LAX. LV SAX (contiguous stack) and LAX (two-chamber and four-chamber) 3T CMR cine images were acquired four times within one scan in 32 women with cardiovascular risk factors (56±10 years, 28±4 kg/m^2^) as follows: 1) resting, gated-segmented, end-expiration breath-hold; 2) resting, real-time, free-breathing; 3) test-retest set of resting, real-time, free-breathing; 4) peak exercise (incremental-to-maximum, in-magnet, stepper test), real-time, free-breathing. A second scan was performed within one week in a subset (n = 5) to determine reproducibility of peak exercise measures. Reliability and agreement of the free-breathing LAX approach with the conventional SAX approach were assessed by intraclass correlation coefficient (ICC) and Bland-Altman plots, respectively. Normal control GLS reserve was also acquired in a separate set of 12 young, healthy control women (25±4 years, 22±2 kg/m^2^) for comparison. Comparisons of LV volumes and function among all techniques at rest had good-to-excellent reliability (ICC = 0.80–0.96), and excellent reliability between peak exercise free-breathing LAX and SAX evaluations (ICC = 0.92–0.96). Higher resting heart rates with free-breathing acquisitions compared to breath-hold (mean difference, limits of agreement: 5, 1–12 beats per minute) reduced reliability for cardiac output (ICC = 0.67–0.79). Reproducibility of the free-breathing LAX approach was good-to-excellent at rest and peak exercise (ICC = 0.74–0.99). GLS exercise reserve was impaired in older women at cardiovascular risk compared to young healthy women (-4.7±2.3% vs -7.4±2.1%, p = 0.001). Real-time, free-breathing CMR with LAX evaluation provides a reliable and reproducible method to assess rest and peak exercise cardiac function, including GLS.

## Background

The study of the mechanisms underlying the spectrum of physical exercise capacity from high performance to disease provides critical insight to our understanding of health, aging, and life span. Reduced exercise capacity is a hallmark of most chronic diseases including cancer, heart disease, lung disease, and diabetes [1–4] due in part, to impaired exercise cardiac function. Performing cardiac imaging combined with exercise enables the quantification of the cardiac determinants of exercise capacity [5] and can also expose occult dysfunction or pathology not apparent at rest [6–8]. Whereas, the standard format of cardiac imaging at rest may miss early or subclinical myocardial impairments due to the heart’s ability to compensate for subtle dysfunction [9]. Accurate, reliable, and reproducible quantification of exercise cardiac function is required to study cardiovascular adaptations in health and disease.

Cardiac magnetic resonance (CMR) imaging is considered the gold standard for quantification of resting cardiac volumes and function based on its high accuracy and reproducibility. Standard resting CMR image acquisition requires breath holds and ECG-gating, both of which are challenging with exercise due to technical and logistical challenges from increased respiratory rates and cardiac translation. To overcome these exercise-specific issues, La Gerche et al. [5] evaluated cardiac volumes and function with an in-scanner exercise stress technique using real-time, free-breathing techniques during submaximal exercise. The conventional contiguous short-axis (SAX) image stack CMR approach was employed with the free-breathing acquisition to quantify exercise cardiac output (CO), and this was validated by the direct Fick method [5]. As an alternative approach, assessing cardiac volumes and function by only long-axis (LAX) imaging offers several advantages. First, LAX evaluation enables the concurrent measurement of left ventricle (LV) global longitudinal strain (GLS), a superior prognostic variable for all-cause and cardiovascular mortality compared to LV ejection fraction when measured at rest in numerous populations [10–12]. Echocardiography-derived exercise reserve in GLS has been shown to be impaired in patients with myocardial impairments [13, 14], and has established normative values [15], but to our knowledge, has not been assessed using CMR. A second advantage of a LAX imaging approach is that the views provide well-defined basal and apical borders of the myocardium, leading to reproducible quantification of LV volumes without the requirement for three-dimensional spatial coverage [16]. This second point is particularly important for accurate exercise volume quantification, due to the large range of cardiac translation (due to high respiratory rates) and the inability to make real-time changes to prescription with CMR. Lastly, a biplane LAX evaluation requires fewer slices (2–6 versus 10–16), and is therefore recommended by the 2020 guidelines for CMR image interpretation and post-processing when rapid acquisition and evaluation are required [17]. However, the reliability and reproducibility of real-time, free-breathing CMR with LAX images, including the assessment of GLS with exercise has not been evaluated.

The primary aim of this study was to describe and evaluate an acquisition and analysis approach using real-time, free-breathing LV biplane LAX images (two-chamber and four-chamber) to measure volumes and function, including GLS at rest and peak in-magnet exercise. First, the reliability and agreement of the real-time, free-breathing LAX (referred to as free-breathing hereafter) approach was compared with the conventional gated-segmented, end-expiration breath-hold (referred to as breath-hold hereafter) acquisition at rest. Second, reliability of the free-breathing LAX approach was compared to the free-breathing SAX approach at rest and at peak in-magnet exercise. Third, reproducibility of the free-breathing LAX approach was evaluated at rest and peak exercise. Lastly, upright cycle ergometer exercise tests were performed to enable the quantification of the extent of exercise stress achieved in-magnet and the cardiac function contributions to VO_2_peak.

## Methods

### Participants

Much of the previous exercise CMR work has been performed in young, healthy/athletic and male only or mixed sex participants [5, 18, 19]. In this study, we enrolled women only as women’s cardiovascular health, especially following breast cancer, is a primary interest of our research group. Thirty-two women with a wide range of cardiovascular risk factors including age, body size, body composition, and physical fitness were prospectively recruited for the dual purposes of assessing reliability and reproducibility of our LAX CMR technique in the current study and as a part of a larger cross-sectional study of anthracycline-treated breast cancer survivors. Inclusion criteria for half of the recruited participants was a history of cardiotoxic treatment for breast cancer, and no history of cancer for the other half. All participants were combined for the purposes of this analysis. Exclusion criteria included contraindications to CMR, common co-morbid conditions that could alter cardiac function (i.e., cardiovascular disease, diabetes mellitus, lung disease). An additional twelve young (22–32 years) women with normal body mass index (19–24.9 kg/m^2^), and no chronic medical conditions (or contraindications to CMR) were recruited to provide normal control data for exercise GLS. All participants were recruited by invitation, word of mouth, and posters in Edmonton, Canada. Ethical approval was obtained from the University of Alberta Research Ethics Board and all participants provided written informed consent.

### CMR acquisition

**Fig 1:**
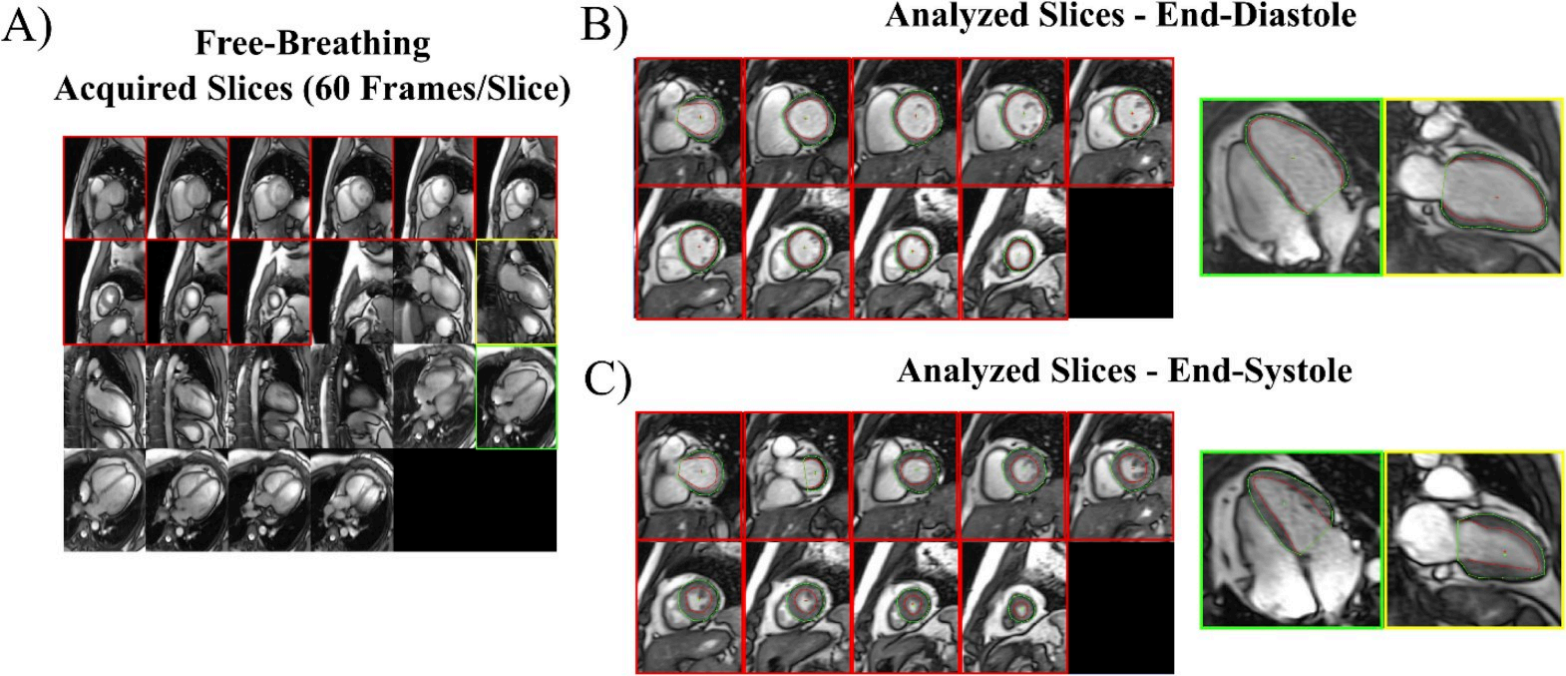
Imaging processing for free-breathing, real-time acquisitions. (A) All slices (10–12 SAX and 6 each of 2 and 4-chamber LAX) are viewed simultaneously for selection of those for analysis. All SAX slices with myocardium and a single 2- and 4-chamber LAX are selected for analysis. A full cardiac cycle for each selected slice is extracted, from which end-diastolic (B) and end-systolic (C) images are identified and endocardial (red) and epicardial (green) borders are traced.

All scans were performed on a 3T Siemens PRISMA system (Siemens Healthcare; Erlangen, Germany) using a 36-element chest/back array for signal reception. Breath-hold acquisitions were performed with ECG-gating at end-expiration and consisted of: 1) segmented contiguous SAX stack of 10–12 slices covering the entire LV; 2) single slices of two-chamber and four-chamber LAX views. Typical imaging parameters were: FOV = 400x300mm, matrix = 256x144, flip angle = 33°, GRAPPA = 3, TE = 1.09ms, TR = 2.38ms, 30 cardiac phases, 8 mm slice thickness, 2 mm gap, 75% phase resolution (true spatial resolution of 1.56 mm x 2.08 mm, interpolated to 0.78 mm x 0.78 mm). Breath-hold, gated imaging was not performed with exercise due to the inability to acquire a usable ECG signal with exercise inside the bore [5] and the challenge of breath holding during high-intensity exercise. Free-breathing acquisitions were acquired real-time at both rest and peak exercise and consisted of: 1) contiguous SAX stack of 12 slices covering the entire LV; 2) six contiguous slices each of the two-chamber and four-chamber LAX views (Fig 1A). Typical imaging parameters were: FOV = 440x260mm, matrix = 224x90, flip angle = 30°, GRAPPA = 3, TE = 1.0ms, TR = 2.12ms, 38ms per image with view sharing, 60 images/slice, 8 mm slice thickness, 2 mm gap, 67% phase resolution (true spatial resolution of 1.96 mm x 2.93 mm, interpolated to 0.98 mm x 0.98 mm). Within the 60 frames acquired, at least two full cardiac cycles were acquired at rest, and on average four full cycles at peak exercise. Complete free-breathing image sets including 12 SAX and 12 LAX slices were acquired in ~50 seconds total.

### CMR protocol

**Fig 2:**
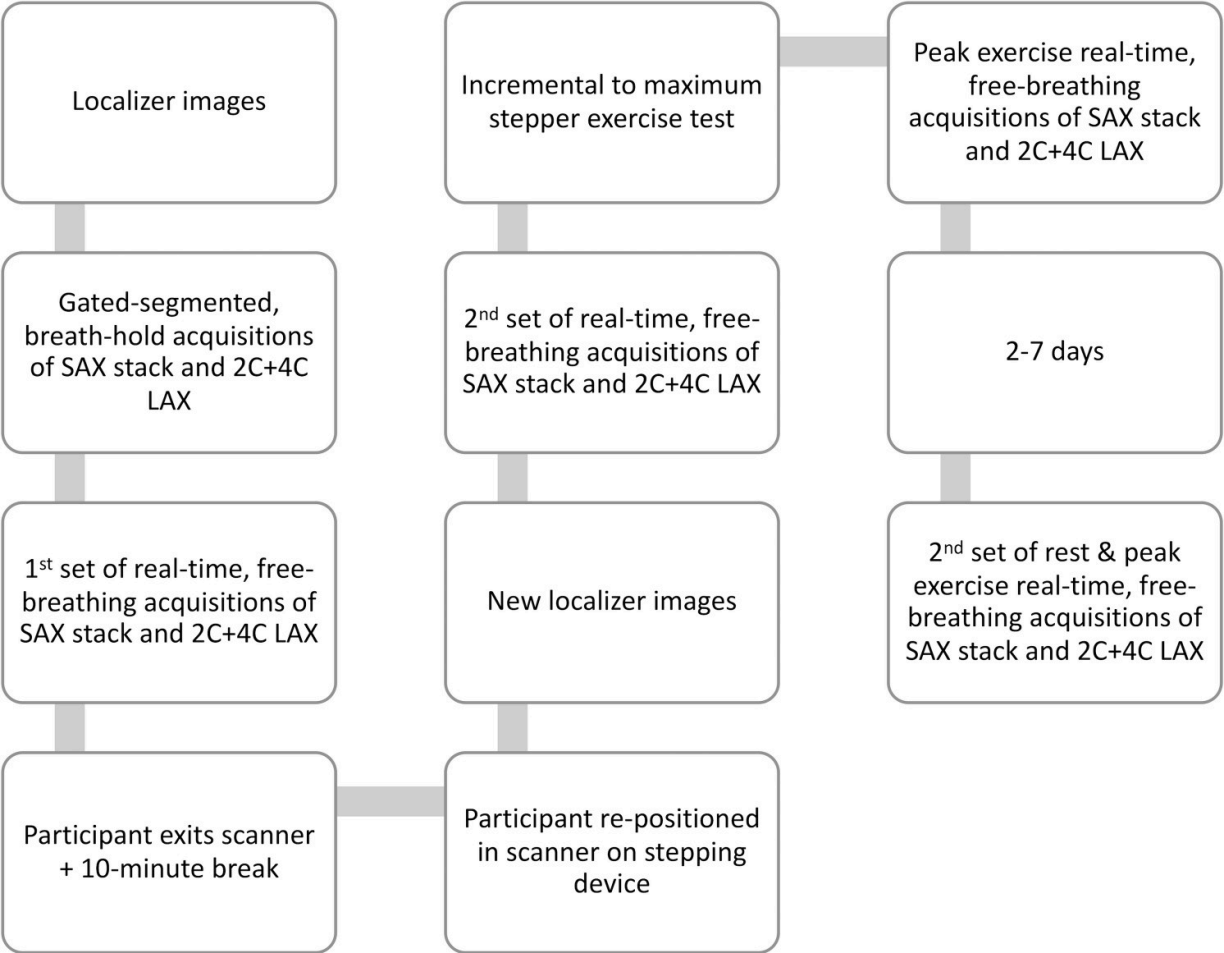
CMR acquisition protocol.

The full CMR protocol is shown in Fig 2. The 32 women recruited for assessing reliability and reproducibility performed the whole CMR and exercise test protocol, while the 12 young controls performed only one full set of the free-breathing rest and peak exercise acquisition, with a subset (n = 5) performing a second full free-breathing set 2–7 days later (as described below).

1. Localizer images were acquired, followed by gated-segmented, breath-hold acquisitions of SAX and LAX images.
2. Free-breathing acquisitions of SAX and LAX images, with matched slice prescriptions to the breath-hold acquisitions above. Participants were instructed to breath gently.
3. The participant then left the MRI scanner for ~10 minutes, and then was repositioned with an MRI-compatible stepping device attached to the table by medical suction (Cardiostep, Ergospect; Innsbruck, Austria). To prevent sliding on the table, the participant wore a snug-fitting vest that was attached to the stepper and their feet were strapped to each step.
4. New localizer images were acquired, followed by a second set of resting free-breathing acquisition of SAX and LAX images to assess reproducibility with 2) above.
5. The exercise protocol was then initiated without removing the participant from the bore. The protocol started with an initial workload of 20 watts and increased by five watts every 20 seconds. An auditory stimulus (beeping) was provided through the patient headphones to help maintain stepping frequency at 40 steps/minute. During the exercise test, a study investigator remained inside the scanner room to provide verbal encouragement and record the participant’s rating of perceived exertion (Borg 0–10 scale) [20]. An MRI-compatible finger pulse oximeter was used to acquire peak heart rate. Free-breathing images were acquired throughout the duration of the exercise to allow adjustments to the prescription to accommodate for cardiac translation due to respiratory depth and body shifting; these images were not used for analysis. The exercise test was terminated when the participant reached volitional exhaustion or could no longer maintain the required stepping frequency. Immediately following the last step at the peak workload, the participant was asked to remain still, and a final complete set of free-breathing SAX and LAX images at peak exercise were acquired.

### CMR analysis

Breath-hold SAX acquisitions were processed using the freely available software Segment version 2.0 (http://segment.heiberg.se) [21]. The Segment automated algorithm was used with manual checking and adjustment for each slice with visible LV for calculation of end-diastolic volume (EDV) and end-systolic volume (ESV) via the method of disks.

For both free-breathing acquisitions (SAX and LAX), image processing with in-house software included: 1) selection of SAX and LAX slices for analysis; 2) identification of a complete cardiac cycle for each slice at similar positions in the respiratory cycle (end-expiration); 3) selection of end-diastolic and end-systolic images; 4) manual tracing of LV endocardial and epicardial contours on end-diastolic and end-systolic images (Fig 1B and 1C). For free-breathing SAX acquisitions at rest and peak exercise, EDV and ESV were calculated using the method of disks, with tracings completed on all slices with visible LV.

For free-breathing LAX acquisitions, EDV and ESV were calculated using the biplane method of disks [22, 23] with tracings completed on a single two-chamber and a single four-chamber image. For all free-breathing analyses, resting heart rate was calculated using the Fourier transform of signal intensity of the LV over all 60 cardiac frames acquired to identify the primary frequency of cardiac motion (i.e., the average duration of the cardiac cycle) (heart rate = 60/average cardiac cycle duration (seconds)).

For all analyses, stroke volume (SV) was measured as the difference between EDV and ESV, and CO measured as the product of SV and heart rate while ejection fraction (EF) was calculated as SV/EDV*100. Additionally, for the breath-hold and free-breathing LAX acquisitions, GLS was calculated as the average of the fractional change in length of the mid-wall contour from end-diastole (L_0_) to end-systole (L) relative to end-diastolic length, reported as a percentage, (L-L_0_)/L_0_*100 (Lagrangian strain) [24] for the two-chamber and four-chamber images, similar to previously reported methods [25]. For both free-breathing evaluations (SAX, LAX), reserve in all parameters was calculated as the difference in each parameter from resting values taken immediately prior to exercise, to peak exercise values.

### Reproducibility of the free-breathing LAX approach

Reproducibility of the free-breathing LAX evaluation approach at rest was assessed from the two sets of acquisitions separated by subject repositioning after leaving the MRI table and a delay of ~10 minutes in the 32 women recruited for reliability and reproducibility. For the evaluation of reproducibility of peak and reserve values, assessments were performed on two different days, separated by 2–7 days, to reduce the impact of fatigue. As the original 32 participants were already performing both the upright cycle ergometer and CMR test within a week, we asked a convenience sample of 5 of the 12 women comprising the young control group to perform the resting assessment and CMR exercise a second time 2–7 days later.

### Comparison to upright exercise responses

The 32 participants involved in the reliability assessment also performed a gold standard upright cycle ergometer (Ergoselect II 1200 Ergoline, Germany) cardiopulmonary exercise test (CPET). The cycle ergometer CPET followed the same protocol as with the stepping device (initial 20 watts workload with a 5 watt increase every 20 seconds until volitional exhaustion). The upright peak power and heart rate were compared to those achieved with the supine, in-magnet, stepper exercise to evaluate the extent of exercise stress. Additionally, oxygen uptake was measured to illustrate the relationship between exercise capacity and CMR-derived CO. Peak oxygen uptake (VO_2_peak) was measured as the highest 20-second VO_2_ captured by continuous gas analysis (Encore229 Vmax; SensorMedics, Yorba Linda, USA). Continuous measurement of heart rate was captured by ECG. The CPET was performed either on a separate day from the MRI scan or on the same day with at least a one-hour break after the MRI exercise test completion according to participant availability and living distance from the testing facility.

### Analysis

Age, anthropometrics, and VO_2_peak values were reported with descriptive statistics, as mean ± standard deviation. Reliability of all parameters was assessed by the intraclass correlation coefficient (ICC) for two-way mixed methods with absolute agreement for the following comparisons between methods: 1) conventional resting breath-hold vs free-breathing LAX; 2) resting free-breathing SAX vs free-breathing LAX; 3) resting free-breathing SAX vs breath-hold SAX; and 4) peak exercise free-breathing LAX vs free-breathing SAX. Agreement was assessed by Bland-Altman plots and the bias and 95% limit of agreement for the free-breathing LAX method with the gold standard (breath-hold SAX for all parameters except GLS which was breath-hold LAX) at rest. The ICC was also calculated to assess reproducibility for our free-breathing LAX method at rest, peak exercise and reserve. Peak power output and heart rate achieved in the CMR exercise test were expressed as a percentage of those achieved in the CPET on the cycle ergometer to assess exercise stress. Linear regression was performed to determine the extent of the variance in VO_2_peak explained by peak CO (calculated by the free-breathing LAX method). Both VO_2_peak and CO were normalized to body weight (kg) to standardize units. GLS exercise reserve was compared between the young normal control data and the 32 participants in the reliability assessment by an independent t-test. SPSS version 26.0 (IBM Corp, Armonk, NY) was used for all analyses.

## Results

### Participants

All 32 participants completed the full CMR examination for the reliability assessment and the CPET. These women had an average ± standard deviation (range) age of 56±10 years (32–72 years), body mass index of 28±4 kg/m^2^ (20–35 kg/m^2^), and VO_2_peak of 26.3±8.2 mL/kg/min (15.4–45.1 mL/kg/min). In terms of cardiovascular risk factors and other medical history, 16 (50%) had a history of cancer and anthracycline treatment, 15 (47%) were former smokers (no current), 14 (44%) were obese, 13 (41%) were overweight, 11 (34%) had arthritis, 6 (19%) were sedentary, 5 (16%) had thyroid disorders, 3 (9%) had osteoporosis, 2 (6%) had hypertension and 2 (6%) had hypercholesterolemia. The twelve women who provided the normal control data for GLS exercise reserve were 25±4 years old with an average body mass index of 22±2 kg/m^2^ and none of the above-mentioned chronic conditions nor any cardiovascular risk factors.

**Fig 3:**
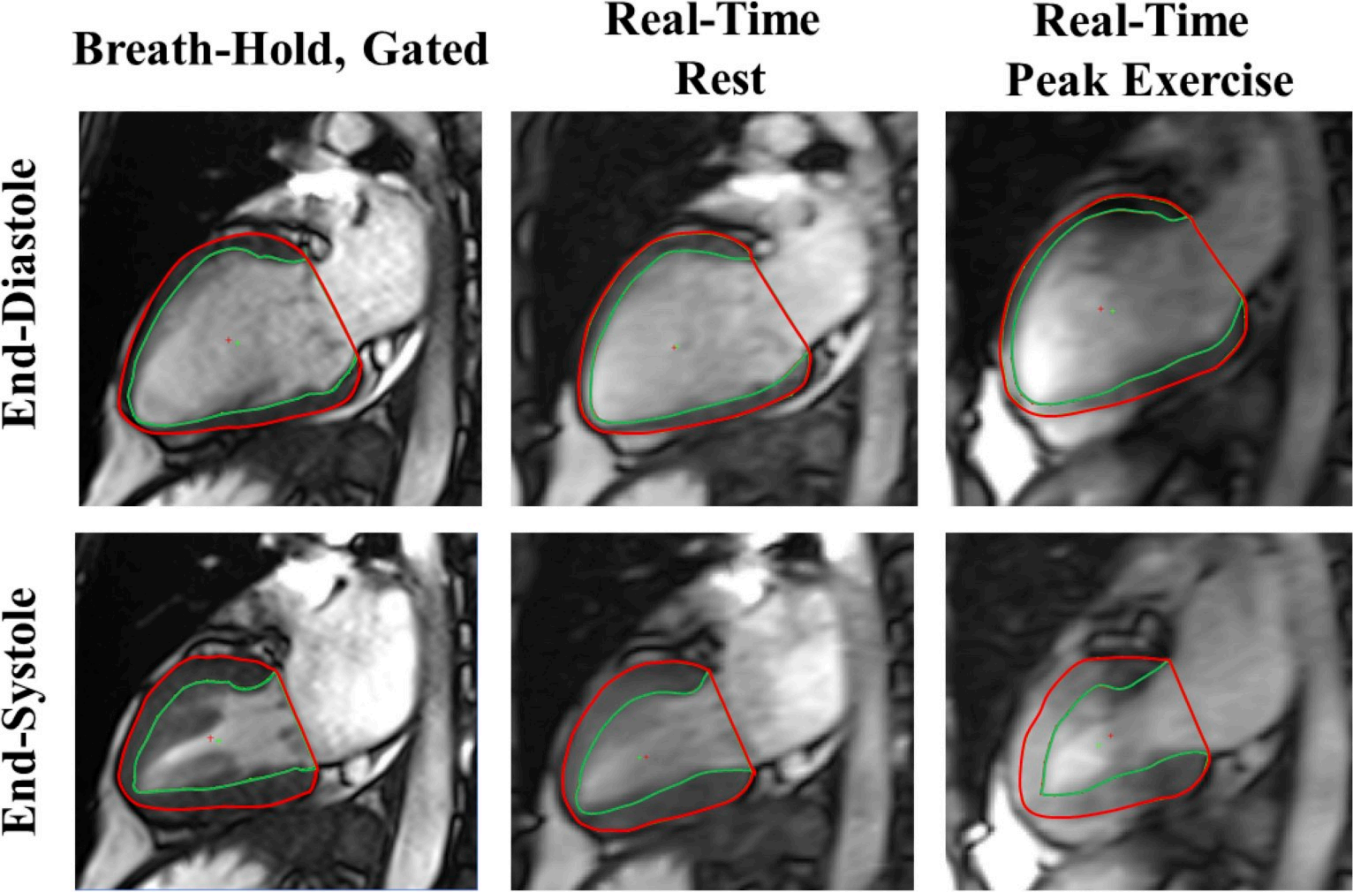
Sample two-chamber long-axis images. Images for the same individual at end-diastole (top row) and end-systole (bottom row) for the conventional gated, end-expiration breath-hold acquisition (first column), real-time, free-breathing acquisition at rest (middle column); and real-time, free-breathing acquisition at peak exercise (right column).

Image quality was sufficient for analysis for all subjects at rest and peak exercise. Fig 3 compares a two-chamber view image analysis for breath-hold and free-breathing scans, at rest and peak exercise.

### Reliability and agreement of free-breathing LAX vs gold standard breath-hold acquisitions at rest

**Fig 4:**
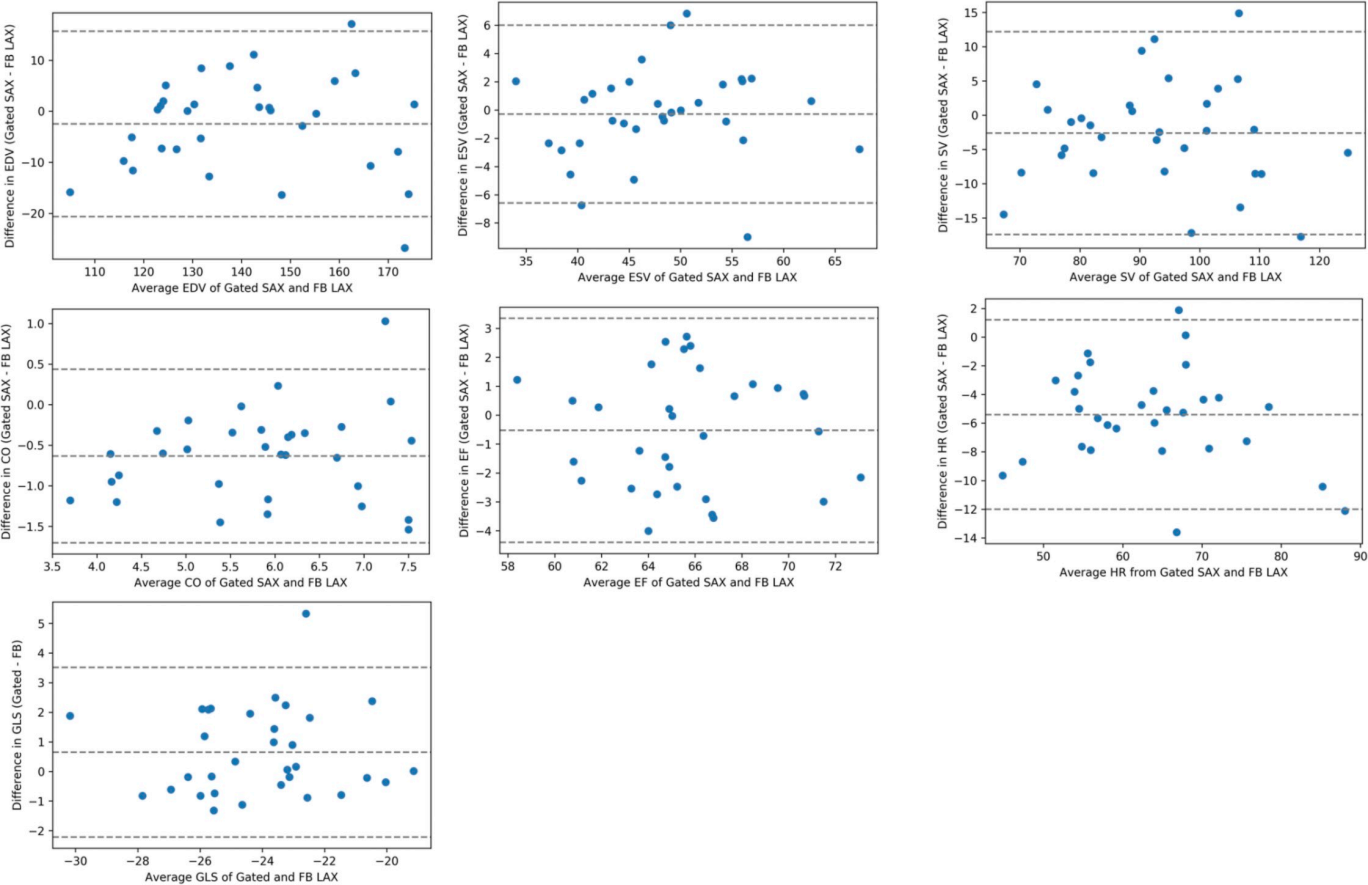
Bland-Altman plots of left ventricular volumes and function and heart rate. Comparison of gold standard gated, breath-hold, short-axis evaluation to a real-time, free-breathing, biplane long-axis evaluation. Dotted lines represent mean difference ±1.96 times the standard deviation of the differences. Abbreviations: EDV, end-diastolic volume; ESV, end-systolic volume; SV, stroke volume; EF, ejection fraction; CO, cardiac output; GLS, global longitudinal strain; HR, heart rate.

At rest, in comparison of the free-breathing LAX approach to the gold standard breath-hold SAX approach, ICC values ranged from 0.76–0.92 for all parameters (Table 1) and Bland-Altman plots illustrate good agreement (Fig 4). Limits of agreement for end-diastolic, end-systolic, and stroke volumes were 16 to -21 mL, 6 to -7 mL, and 12 to -17 mL, respectively, with negligible (0–3 mL) mean differences. The mean difference in ejection fraction was -1% and the limits of agreement were 3 to -4%. In comparison of the free-breathing and breath-hold GLS (-24.4±2.5% vs -23.7±2.5%), agreement was good (ICC = 0.80), but Bland-Altman plots demonstrated that the free-breathing technique resulted in a slight overestimation with a mean difference of 1.0% and limits of agreement of 4.2 to -2.1% (Fig 4).

**Table 1:** Free-breathing LAX acquisitions versus breath-hold and free-breathing SAX acquisitions for resting LV volumes and function, and heart rate.

Parameter	SAX breath-hold	LAX free-breathing	SAX free-breathing	Breath-hold SAX vs free-breathing LAX	Free-breathing SAX vs free-breathing LAX	Breath-hold SAX vs free-breathing SAX
End-diastolic (mL)	140±20	142±20	139±20	0.89	0.93	0.88
End-systolic volume (mL)	48±8	48±8	47±8	0.92	0.96	0.92
Stroke volume (mL)	92±15	94±15	92±15	0.84	0.91	0.84
Ejection fraction (%)	65±4	66±4	66±4	0.83	0.92	0.80
Cardiac output (L/min)	5.5±1.2	6.2±1.1	6.1±1.2	0.76	0.79	0.67
Heart rate (bpm)	61±10	66±11	67±11	0.83	0.85	0.71

Data are mean ± standard deviation. Abbreviations: SAX, short-axis; LAX, long-axis; LV; left ventricular; ICC, intraclass correlation; GLS, global longitudinal strain.

Both resting heart rate and thus CO were higher with free-breathing compared to breath-hold acquisitions (Table 1). Bland-Altman plots demonstrate higher heart rates, 5 beats per minute (bpm) higher, on average, compared to the breath-hold acquisition (Fig 4). The resulting free-breathing CO was also higher, with a mean difference of 0.63 L/min, and limits of agreement from -1.7 to 0.4 mL (Fig 4).

We also compared the free-breathing SAX acquisitions to the conventional breath-hold SAX acquisitions which had good to excellent reliability (ICC values ranged from 0.80 to 0.92).

### Reliability of free-breathing LAX vs SAX evaluations at rest and peak exercise

At rest and peak exercise, ICC values for comparing the LAX and SAX free-breathing acquisitions exceeded 0.90 for all parameters (Tables 1 and 2, respectively). ICC values for reserve values ranged from 0.43 to 0.91 (Table 2).

**Table 2:** SAX versus LAX real-time free-breathing approaches for peak exercise and reserve LV volumes and function.

Parameter	Exercise Values		Reserve Values (Exercise–Rest)		ICC Comparison	
	LAX	SAX	LAX	SAX	Exercise LAX vs SAX	Reserve LAX vs SAX
End-diastolic (mL)	149±25	147±23	6±10	7±11	0.94	0.55
End-systolic volume (mL)	38±9	38±9	-11±6	-9±6	0.96	0.82
Stroke volume (mL)	111±19	109±18	16±8	16±9	0.92	0.43
Ejection fraction (%)	75±4	74±4	9±3	8±3	0.94	0.74
Cardiac output (L/min)	15.5±3.3	15.2±2.9	9.4±2.6	9.0±2.4	0.94	0.90
Heart rate (bpm)	140±17	140±17	74±16	73±17	-	0.93
GLS (%)	-29.2±2.6	-	-4.7±2.3	-	-	-

Data are mean ± standard deviation. Abbreviations: SAX, short-axis; LAX, long-axis; LV, left ventricular; ICC, intraclass correlation; GLS, global longitudinal strain.

### Reproducibility of free-breathing LAX approach

ICC values for reproducibility for LV volumes and function at rest and peak exercise ranged from 0.74 to 0.99 (Table 3). ICC values for reserve in all parameters ranged from 0.64 to 0.95 (Table 3).

**Table 3:** Test-retest reliability by intraclass correlation of real-time, free-breathing, long-axis evaluation approach.

Parameter	Resting	Peak Exercise	Reserve
End-diastolic volume	0.93	0.99	0.73
End-systolic volume	0.89	0.92	0.70
Stroke volume	0.88	0.97	0.67
Ejection fraction	0.74	0.87	0.64
Cardiac output	0.76	0.97	0.96
Heart rate	0.96	0.88	0.95
Global longitudinal strain	0.79	0.91	0.69

### Comparison to upright exercise responses

**Fig 5:**
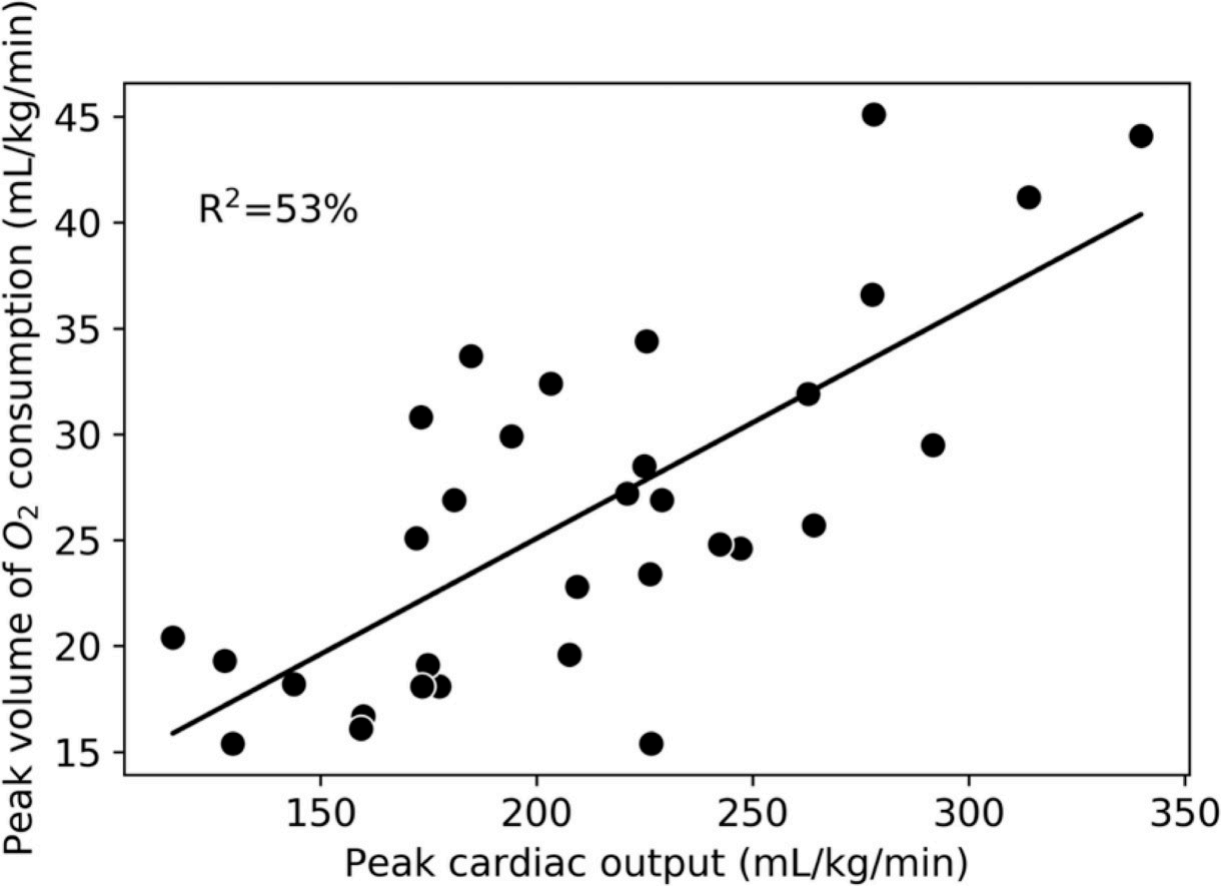
Linear relationship between peak cardiac output and peak volume of oxygen consumption. Cardiac output measured via long-axis, real-time, free-breathing acquisition. Both variables are normalized to body weight.

On the upright cycle ergometer test, the objective criteria of achievement of VO_2_peak, a respiratory exchange ratio exceeding 1.1 [26], and volitional exhaustion were achieved in ≥98% of tests. On the CMR exercise test, all participants reported a peak rating of perceived exertion of ≥9 on the Borg 0–10 scale. The average peak power output and heart rate achieved on the cycle ergometer test were 148±34 watts and 164±13 bpm. In comparison, on the supine CMR stepper exercise test, the average peak values achieved were 137±32 watts and 142±15 bpm, which corresponds to 93±11% and 86±7% of those on the upright cycle ergometer. LAX peak CO explained 53% of the variance in VO_2_peak (Fig 5).

### CMR-derived GLS exercise reserve normal control values in women

The GLS exercise reserve in the normal control group was a mean±standard deviation of -7.4±2.1 percentage points with a range of -3.5 to -10.9 percentage points. The reserve was significantly higher than in the 32 women used in the reliability analysis (-4.7±2.3 percentage points, p = 0.001).

## Discussion

In this study, we described and evaluated an approach for the quantification of both resting and peak exercise LV volumes and function, including GLS, using real-time, free-breathing biplanar LAX acquisitions. We demonstrated that at rest, this approach had very good to excellent reliability and agreement with the gold standard, gated-segmented, end-expiration breath-hold, SAX evaluations for all parameters. At peak exercise, the free-breathing LAX approach was shown to have excellent reliability compared to a free-breathing SAX approach that has been previously validated against the direct Fick method for quantification of CO [5]. Reproducibility of the free-breathing LAX approach was good to excellent at rest and peak exercise. This approach was evaluated in women with a wide range of age, body size, and fitness levels, who achieved 93% of peak upright cycle ergometer exercise workload, with successful imaging evaluations in all cases.

### LAX CMR

The LAX approach to analyzing LV volumes and function offers the advantage of analysis of only two slices, without the requirement of a large number of respiratory-phase matched contiguous slices to generate a volume, as needed for SAX analysis [5]. Gated-segmented, breath-hold LAX CMR approaches have previously been evaluated as an alternative to standard full volumetric coverage with SAX imaging. Bloomgarden et al. [27] compared conventional SAX CMR values to a LAX approach consisting of 6 to 9 radial LAX slices, reporting similar ICC values for resting LV volumes and function (0.75–0.97) as the current study. Childs et al. [16] reported higher inter-observer reliability with LAX compared to SAX CMR evaluations with the use of 6 radial long-axis slices. Huttin et al. [28] compared a biplane LAX evaluation to a conventional SAX approach in patients with acute myocardial infarction, showing higher inter-observer and intra-observer reliability with the LAX approach. We extended these findings in the present study by including a real-time, free-breathing biplane LAX approach, showing similarity of all volumetric and functional parameters to both gated and free-breathing SAX analyses, as well as demonstration of good to excellent reproducibility for rest and peak exercise. To our knowledge, this is the first report of reliability of a CMR LAX approach with exercise challenge.

A unique aspect of the LAX approach in the current study was the acquisition of six parallel LAX slices to enable retrospective selection of optimally placed mid-ventricular LAX slices (one two-chamber and one four-chamber view). This technique helped to account for cardiac translation during exercise. The total acquisition time in this study for SAX and LAX slices were similar (~25 seconds each), but the LAX acquisition could be reduced to ~13 seconds with the acquisition of just three (instead of six) contiguous slices each view, which in our experience would be sufficient to account for translation. This is significantly shorter than the conventional technique (requiring gating, breath-holds for 12 SAX slices), which would take 5–7 minutes depending on heart rate and recovery time between breath-holds. However, a limitation of this approach is that without the 3-chamber LAX view, the regional wall motion in the anteroseptum and inferior segments cannot be assessed.

### Global longitudinal strain

A primary motivation to using a LAX approach is the concurrent ability to measure GLS. Resting GLS has been demonstrated to be a strong independent predictor of cardiac events, cardiac mortality and all-cause mortality [10–12, 29]; notably the predictive value for all-cause mortality is nearly two-fold greater than LVEF [12]. LVEF is a global measure that, when measured at rest, can be insensitive to subtle myocardial damage due to the myocardium’s considerable recruitable contractile ability to compensate. In contrast, GLS has been shown to be more sensitive than LVEF as exemplified by the well-established application of GLS for early detection of impaired cardiac function associated with cardiotoxic cancer therapy [29].

Peak exercise or reserve of LVEF and GLS could augment sensitivity of the detection of dysfunction over imaging performed at rest. For example, impaired LVEF reserve after just two anthracycline treatments, was shown to predict later development of cardiac dysfunction [30] and GLS reserve is impaired among patients with cardiac conditions (mitral regurgitation, aortic stenosis) compared to controls [13, 14]. GLS reserve in the current study was higher among young healthy women (-7.4±2.1%) than for the women in the reliability assessment group who were older and had a number of cardiovascular risk factors but no overt cardiac disease (4.7±2.3%). To our knowledge, prior studies measuring peak or reserve GLS have primarily used echocardiography [14, 31, 32]. Our average exercise CMR-derived GLS reserve for young healthy women (-7.4±2.1%) is higher than values reported for exercise echocardiography (5.2±1.7%) [14] and adenosine echocardiography (5.0±3.2%) [30], among mixed age and gender, non-diseased controls.

### Heart rate

While CMR LV volumes and function were similar for all compared approaches, resting heart rate measured during free-breathing acquisitions was 5 bpm greater than reported with gated, breath-hold acquisitions. This finding is consistent with a previous report of a 4 bpm drop in heart rate during breath holding [33]. CO measured with breath-hold acquisitions was thus correspondingly lower due to heart rate, as volumes were similar to free-breathing evaluations. Therefore, CO measured with free-breathing acquisition is likely more representative of the true baseline for comparison with free-breathing exercise values for calculation of reserve.

#### CMR exercise

One aim of this study was to evaluate the extent of exercise stress achieved with the supine MRI stepper device compared to a CPET performed on an upright cycle ergometer. The supine body position required for CMR exercise is expected to lead to alternations in cardiovascular parameters compared to an elevated or upright position, including a lower heart rate, increased LV filling or EDV, and increased SV [34]. In this study, the peak heart rate was 15% lower on average in the supine position compared to the upright exercise. La Gerche et al. [5] determined in pilot work among 15 healthy volunteers (13 males) that 66% of upright cycle ergometer power corresponded to maximal supine exercise and subsequently used this workload in other studies of exercise CMR [35]. Le et al. [36] found that maximal supine cycle ergometer power corresponded to 80% of upright power in athletes and healthy volunteers. In contrast, in our study, women with lower absolute peak power outputs than these studies achieved 93±11% of upright cycle ergometer peak power output in maximal supine exercise within the MRI bore. Differences in sex, peak power output or our exercise protocol (especially having a tester remain in the scanner room for motivation) could all contribute to this discrepancy.

The higher relative intensity achieved in our study has implications. First, it illustrates the feasibility of maximal exercise evaluations with CMR imaging in women with a wide range of ages, body sizes, and fitness levels. Second, cardiac function parameters measured at a higher intensity are likely more representative of cardiac function at peak exercise in an upright position. Lastly, the peak power output achieved with the supine stepper device could be taken to be representative of fitness levels, thereby potentially removing the requirement for a second exercise test in some studies.

Peak exercise CO is one of the primary determinants of VO_2_peak, and increases with exercise intensity determined by both heart rate and SV. In this study, the increase in SV was due primarily to an exercise-related decrease in ESV. EDV changed minimally with exercise due to the supine posture, which is similar to previous reports [19]. Over half of the variance in upright VO_2_peak was explained by our LAX CMR technique for assessing supine peak CO. The Fick equation states that the other determinants of VO_2_peak include hemoglobin concentration and arteriovenous oxygen difference [37]. While Fig 5 demonstrates a linear relationship between CO and VO_2_peak, it is also evident that substantial variation in peak CO exists among participants with very similar VO_2_peak values. This finding applies across the spectrum of fitness levels measured in this study. This data could be used to determine the extent of central versus peripheral limitations to fitness within individual participants and personalize approaches to improve those limitations [37].

### Limitations

We did not compare our free-breathing LAX approach to direct evaluations of cardiac function (i.e., direct Fick method for calculation of CO). However, a free-breathing SAX approach has been previously validated in this way, and our free-breathing LAX data showed excellent agreement with our free-breathing SAX data. Respiration is known to influence cardiac volumes but we did not systematically collect information on the timing of respiration as has been done by others by measuring abdominal pressure with a plethysmograph [35]. However, the use of visual selection of SAX images at end-expiration resulted in acceptable variation as demonstrated by our high agreement between free-breathing and breath-hold acquisitions. In order to acquire images fast as possible without a reduction in spatial resolution, we did not adjust temporal resolution for higher heart rates, which could result in not capturing the true end-systolic volume at peak exercise. Our test-retest reliability assessment of peak exercise values was limited to only five cases due to costs and logistics, and this may overestimate the true reliability. Our study sample did not include individuals with significant cardiac dysfunction such as ischemic disease or abnormalities where the geometric assumptions of the LAX approach would be limiting [38]. Accurate prescription of the LAX slices is required to prevent foreshortening thus, it may be at a higher risk for inaccuracy than the SAX approach in inexperienced technicians. However, acquisition of several parallel LAX slices enables retrospective selection of mid-ventricular slices, with the largest cross-sectional areas, thus minimizing sensitivity to slice prescriptions.

## Conclusion

A real-time, free-breathing CMR approach to measure rest and peak exercise cardiac function using two-chamber and four-chamber view LAX images demonstrated good to excellent absolute agreement, with no major systematic differences from the gold standard approach of gated, breath-hold SAX evaluation of LV volumes and function at rest as well as free-breathing SAX evaluation at rest and peak exercise. Test-retest reliability of this approach was very good to excellent at rest and peak exercise. Overall, this real-time, free-breathing LAX CMR technique provides an accurate and reliable method to assess rest and peak cardiac function and could be used to expose clinical abnormalities that may be unidentifiable at rest and assess the contributions of cardiac function to exercise capacity.
